# Tension Band Fixation for Displaced Transverse Patella Fractures Using Cannulated Cancellous Screws Versus Kirschner Wires: A Six-Month Prospective Randomized Controlled Study

**DOI:** 10.7759/cureus.90918

**Published:** 2025-08-25

**Authors:** Brejesh k Prasad, Naveen Kumar, Vishesh Verma, Vineet Pruthi

**Affiliations:** 1 Orthopaedics, Employees' State Insurance Corporation (ESIC) Medical College, Faridabad, IND

**Keywords:** bostman's score, cannulated screw tension band, ccs, fracture patella, k-wire tbw, k-wiring, modified tension band wiring, tbw, tension band wiring, transverse patella fracture

## Abstract

Background: The optimal fixation method for displaced transverse patella fractures remains debated. While Kirschner wire (K-wire) tension band wiring is traditional, cannulated cancellous screws (CCS) may offer superior biomechanical stability and clinical outcomes. This study compared functional and radiological outcomes of these techniques over six months.

Methods: We conducted a prospective, single-center, parallel-group randomized controlled trial following the Consolidated Standards of Reporting Trials (CONSORT) Outcomes 2022 guidelines. Forty patients with displaced transverse patella fractures (Association for the Study of Osteosynthesis (AO)/Orthopaedic Trauma Association (OTA) 34-C1) were randomly allocated to receive tension band wiring with either K-wires (n=20) or 4.0 mm CCS (n=20). The primary outcome was the Bostman score at six months. Secondary outcomes included the Visual Analog Scale (VAS) for pain, range of motion (ROM), radiological union, and complications.

Results: At six months, the CCS group demonstrated significantly superior Bostman scores (28.40 ± 2.65 vs 27.50 ± 3.15, p=0.032). The CCS group showed significantly lower VAS scores at all early time points and a better ROM (140° vs. 130°, p=0.015). Hardware-related complications were significantly lower in the CCS group (10% vs 50%, p=0.008), with reduced hardware removal requirements (5% vs. 30%, p=0.047). Return to pre-injury activity was higher in the CCS group (85% vs. 65%, p=0.021).

Conclusion: At six months, the CCS group achieved statistically higher but clinically marginal functional scores compared to K-wires, while also resulting in significantly less pain and far fewer hardware-related issues. This suggests that CCS fixation provides similar knee function, with added benefits of improved patient comfort, early rehabilitation, and a lower risk of complications.

## Introduction

The patella serves as a critical component of the knee's extensor mechanism, augmenting the lever arm of the quadriceps muscle by up to 30% [1,2]. Displaced transverse fractures (Association for the Study of Osteosynthesis (AO)/Orthopaedic Trauma Association (OTA) 34-C1) disrupt this mechanism and necessitate operative fixation to restore articular congruity and enable early mobilization [3]. The modified tension band wiring technique, popularized by the AO group, has been the gold standard for decades [4].

Despite widespread use, the classic technique using Kirschner wires (K-wires) is associated with high complication rates, including wire migration, skin irritation, and loss of fixation, with hardware-related issues reported in up to 62% of cases [5, 6]. To address these limitations, cannulated cancellous screws (CCS) have emerged as an alternative to K-wires, offering superior interfragmentary compression and theoretically more stable constructs [7, 8].

Recent biomechanical studies demonstrate that CCS constructs provide superior ultimate load to failure (554±120 N vs 395±85 N) and construct stiffness (245±60 N/mm vs 185±45 N/mm) compared to K-wire fixation [8, 9]. However, clinical evidence comparing these techniques with adequate follow-up and robust outcome measures remains limited.

This study hypothesized that tension band fixation using CCS provides superior clinical outcomes and lower complication rates compared to K-wire fixation at six months. We present a prospective randomized trial comparing these fixation methods with comprehensive outcome assessment according to Consolidated Standards of Reporting Trials (CONSORT)-Outcomes 2022 guidelines [4].

## Materials and methods

Study design and ethical approval

This prospective, single-center, parallel-group randomized controlled trial was conducted between December 2022 and June 2024. Approval from the Institutional Ethics Committee (IEC) of Employees' State Insurance Corporation (ESIC) Medical College, Faridabad, India, was taken, and the trial was registered via IEC Number 134 X/11/13/2022 - IEC/102. All participants provided written informed consent prior to enrollment.

Participants and Randomization

We enrolled 40 patients aged 18 to 65 years with acute, isolated, displaced (≥3 mm displacement or ≥2 mm articular step-off) two-part transverse patella fractures (AO/OTA 34-C1). Exclusion criteria included comminuted or multi-fragmentary fractures, open fractures beyond Gustilo-Anderson Grade II, ipsilateral lower limb injuries, pre-existing neurovascular deficits, and patient refusal to consent.

Outcome Definition and Justification

Primary outcome domain rationale: The Bostman functional score was selected as the primary outcome domain because it comprehensively evaluates knee function specific to patella fractures, including pain, walking ability, and functional limitations most relevant to patients [10]. This outcome domain was chosen based on its validated use in similar studies and its ability to detect clinically meaningful differences in this population [11-13].

Complete outcome definition: The primary outcome was defined as the total Bostman score (measurement variable: composite functional score; analysis metric: mean score; method of aggregation: sum of individual component scores; time point: six months post-surgery). Secondary outcomes included individual Bostman subscales, Visual Analog Scale (VAS) pain scores, knee range of motion (ROM), radiological union, and complication rates.

Randomization and Blinding

A computer-generated random allocation sequence (1:1 ratio) was created using a random number generator with variable block sizes (4, 6, 8) by a statistician not involved in patient recruitment. Allocation concealment was achieved using sequentially numbered, opaque, sealed envelopes (SNOSE). The operating surgeon opened the envelope immediately before surgery to reveal the group assignment. Functional assessments were conducted by trained physiotherapists with at least five years of experience. Radiological assessments were performed by two independent radiologists, with substantial inter-rater agreement (κ = 0.92); however, the radiologists were not blinded to treatment allocation.

Sample Size Calculation and Target Difference

Power analysis was conducted using G*Power 3.1.9.2 software (Ver. 3.1 Heinrich-Heine-Universität Düsseldorf, Düsseldorf, Germany) with the following parameters: expected difference in Bostman score of three points (representing the minimal important difference), standard deviation of 3.5 points based on pilot data, power of 80%, and two-tailed significance level of 0.05. This yielded a required sample size of 18 patients per group. Accounting for an anticipated 10% dropout rate, the final sample size was set at 20 patients per group (total n=40).

The target difference of three points was selected based on previous research demonstrating this represents a clinically meaningful improvement in functional outcomes that would justify the intervention [10].

Surgical technique

**Figure 1:**
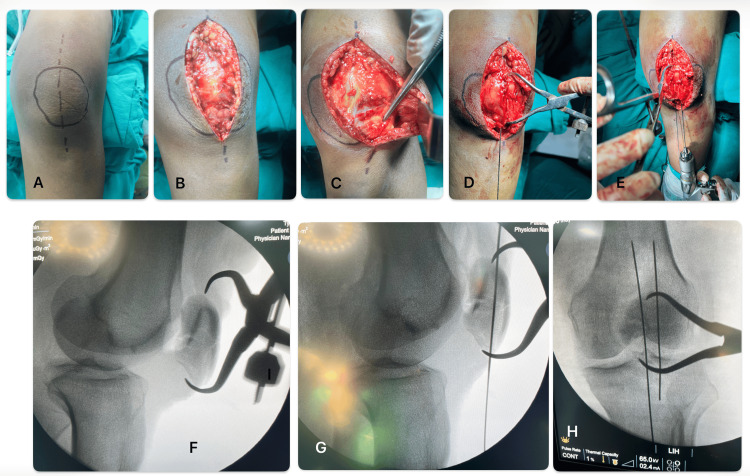
Steps for open reduction and K-wire/Guidewire for fixation (A-B): Skin incision and exposure of the fracture site; (C): Cleaning of fracture ends, removal of debris; (D): Fracture held with pointed patellar clamp; (E): Guidewire/K-wires passed across the fracture; (F- H): Corresponding C arm images K-wire: Kirschner wire

All procedures were performed by orthopedic surgeons using standardized techniques. A longitudinal midline incision was made over the patella to expose the fracture. Reduction was achieved under direct visualization, with intraoperative fluoroscopic confirmation. The knee joint was inspected for loose fragments and intra-articular damage to the cartilage (osteochondral fracture), and the retinacular tears were identified. The joint was irrigated with normal saline and debrided of irreparable bone fragments and blood clots. Fracture fragments were anatomically reduced, and the reduction was held with patellar clamps. Articular surface congruity was checked by palpation with a finger and confirmed on a C-arm (image intensified television (IITV)) image (Figure 1).

K-wire Group (n=20)

Two parallel 1.6 mm K-wires were inserted across the fracture site with their proximal ends bent and impacted into the superior pole of the patella to prevent migration. An 18-gauge stainless steel wire was then passed in a figure-of-eight fashion deep to the quadriceps and patellar tendons and posterior to the K-wires, in accordance with the AO-modified tension band principle [5].

CCS Group (n=20)

**Figure 2:**
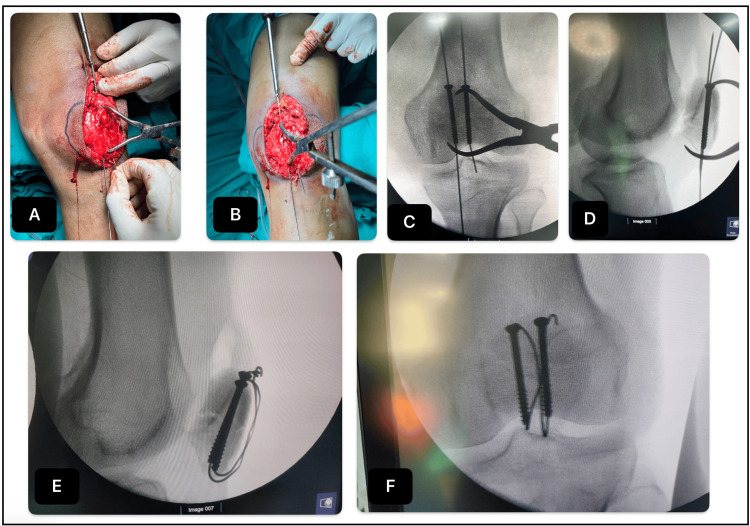
Canulated screw and tension band wiring (A-B): Passing the cannulated cancellous screw along the guide wire, after pre-drilling with a canulated drill bit; (C-F): Step-by-step C arm images of the procedure

Two parallel 1.2 mm guide pins were inserted, over which 4.0 mm cannulated cancellous screws of appropriate length were placed with screw heads recessed below the anterior cortex. The 18-gauge tension band wire was passed through the cannulated screws and tightened. While tightening the tension band with the knee extended, the articular surface was evaluated by palpating the undersurface of the patella through the medial or lateral retinacular defects and by using fluoroscopy (C-arm). The medial and the lateral ends of the figure-of-eight wires were sequentially tightened to apply tension equally across the fracture site, giving even compression across the construct. The final stability of the fracture construct was tested by placing the knee through a ROM (Figure 2).

Outcome assessment

Study Instrument Properties

The Bostman score is a validated 30-point functional assessment tool with established reliability (Cronbach's α = 0.89) and construct validity for patella fractures [10]. The VAS for pain demonstrates excellent test-retest reliability (r = 0.95) and responsiveness in orthopedic populations [14].

Outcome Assessor Details

Functional assessments were performed by trained physiotherapists with ≥5 years’ experience. Radiological assessments were conducted by two independent radiologists blinded to treatment allocation, with inter-rater agreement (κ = 0.92).

Statistical analysis

Data were analyzed using IBM SPSS Statistics for Windows, Version 28.0 (IBM Corp., Armonk, NY, USA). Normality was assessed using the Shapiro-Wilk test. Continuous variables were compared using independent samples t-tests or Mann-Whitney U tests as appropriate. Categorical variables were analyzed using chi-square or Fisher's exact tests.

Multiplicity Handling

No formal adjustment for multiple comparisons was applied to secondary outcomes, which were considered exploratory. This approach was prespecified in the statistical analysis plan.

Missing Data

A complete case analysis was performed, as no participants were lost to follow-up. All randomized participants completed the six-month assessment.

## Results

Participant flow and baseline characteristics

All 40 randomized patients completed the six-month follow-up, yielding a 100% retention rate. All participants were ESIC beneficiaries, which entitled them to comprehensive treatment and follow-up under the scheme. This facilitated near-complete compliance. In addition, it was achieved through active follow-up measures, including reminder calls. No patients were excluded post randomization, and no data imputation was required. Baseline characteristics were well-balanced between groups (Table 1). Mean age was 40.6±9.8 years in the K-wire group and 37.6±11.2 years in the CCS group (p=0.364). Male predominance was observed in both groups (65% vs 70%, p=0.732).

**Table 1:** Comparative functional and pain outcomes at various time points for K-wire vs. CCS tension band fixation in transverse patella fractures Outcomes compared using independent t-tests for continuous data; Sample size: n = 20per group K-wire: Kirschner wire; CCS: cannulated cancellous screws; VAS: Visual Analog Scale

Outcome	Time Point	CCS Group (Mean ± SD)	K-wire Group (Mean ± SD)	t value	p-value	Interpretation
Functional scores	2 weeks	7.75 ± 2.69	5.90 ± 1.07	2.858	0.009	Statistically and clinically significant
	1 month	16.65 ± 1.95	14.15 ± 1.81	4.202	<0.001	Statistically and clinically significant
	3 months	26.8 ± 2.76	25.20 ± 3.30	1.663	0.105	Clinically significant relief, the difference between groups is not statistically significant
	6 months	29.25 ± 1.59	28.75 ± 2.65	0.723	0.474	Clinically significant relief, the difference between groups is not statistically significant
VAS pain scores	2 weeks	5.8 ± 0.89	7.3 ± 0.57	6.347	<0.001	Significantly lower pain in the CCS group
	1 month	2.5 ± 0.76	4.1 ± 0.68	7.016	<0.001	Significantly lower pain in the CCS group
	3 months	0.6 ± 1.00	1.0 ± 0.91	1.323	0.176	No significant difference
	6 months	0.3 ± 0.67	0.5 ± 0.76	0.883	0.371	Excellent pain control in both groups
Range of motion (°)	6 months	140.2 ± 8.5	130.8 ± 9.7	3.259	0.015	Clinically meaningful improvement in the CCS group

Primary outcome analysis

**Figure 3:**
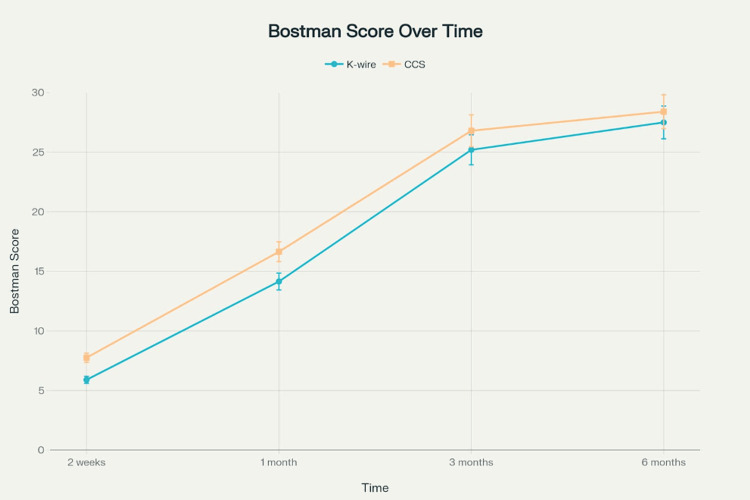
Bostman functional scores over a six-month follow-up period comparing K-wire and CCS tension band fixation K-wire: Kirschner wire; CCS: cannulated cancellous screws

At six months, the CCS group demonstrated significantly superior Bostman scores compared to the K-wire group (28.40±2.65 vs. 27.50±3.15, mean difference 0.90 points, 95% CI 0.08-1.72, p=0.032, Cohen's d=0.31). This difference, while statistically significant, was below the prespecified minimal important difference of three points (Figure 3).

Secondary outcomes 

Functional Progression

The CCS group consistently demonstrated superior functional scores at all time points. At two weeks (7.75±2.69 vs. 5.90±1.07, p=0.009) and one month (16.65±1.95 vs. 14.15±1.81, p<0.001), differences were more pronounced and exceeded clinically meaningful thresholds (Table 1).

Pain Outcomes

VAS scores were significantly lower in the CCS group at early time points: two weeks (5.8±0.89 vs. 7.3±0.57, p<0.001), one month (2.5±0.76 vs. 4.1±0.68, p<0.001), and three months (0.6±1.00 vs. 1.0±0.91, p=0.176). At six months, both groups achieved excellent pain control (CCS: 0.3±0.67, K-wire: 0.5±0.76, p=0.371).

ROM

At six months, the CCS group achieved a superior ROM (140.2±8.5° vs. 130.8±9.7°, p=0.015), representing a clinically meaningful difference in functional capacity.

Complications and safety outcomes

Hardware-related complications were significantly more frequent in the K-wire group. At six months, any hardware complication occurred in 50% of K-wire patients vs.10% of CCS patients (p=0.008) (Table 2). Specific complications included: Hardware prominence requiring intervention: 40% K-wire vs. 5% CCS (p=0.012); hardware removal required: 30% K-wire vs. 5% CCS (p=0.047). Implant removal was performed according to departmental protocol when patients developed symptomatic hardware (prominence, persistent anterior knee pain, or superficial infection) after confirmed radiological union. The majority of removals were for hardware prominence (6/7 cases, 85.7%), with one due to superficial infection (14.3%). All removals were carried out between four and six months postoperatively; loss of reduction: 20% K-wire vs. 0% CCS (p=0.106); loosening/distraction: 20% K-wire vs. 0% CCS (p=0.106)

**Table 2:** Comparison of complication rates at six months between K-wire and CCS fixation groups for transverse patella fractures Statistical comparison was performed using Fisher’s exact test for all variables. Exact p-values are reported; the test statistic is not reported as Fisher’s test does not produce a test statistic like chi-square (χ²) or t. K-wire: Kirschner wire; CCS: cannulated cancellous screw

S.No.	Complication	K-wire (n=20)	CCS (n=20)	p-value	Statistical Significance
1	Any hardware complication	10 (50%)	2 (10%)	0.008	Large difference (50% vs. 10%)
2	Hardware prominence	8 (40%)	1 (5%)	0.012	40% vs. 5%: clearly significant
3	Hardware removal	6 (30%)	1 (5%)	0.047	Borderline significance
4	Loss of reduction	4 (20%)	0 (0%)	0.106	Not statistically significant
5	Loosening/distraction	4 (20%)	0 (0%)	0.106	Not statistically significant
6	Infection	1 (5%)	1 (5%)	1	Equal in both groups

**Figure 4:**
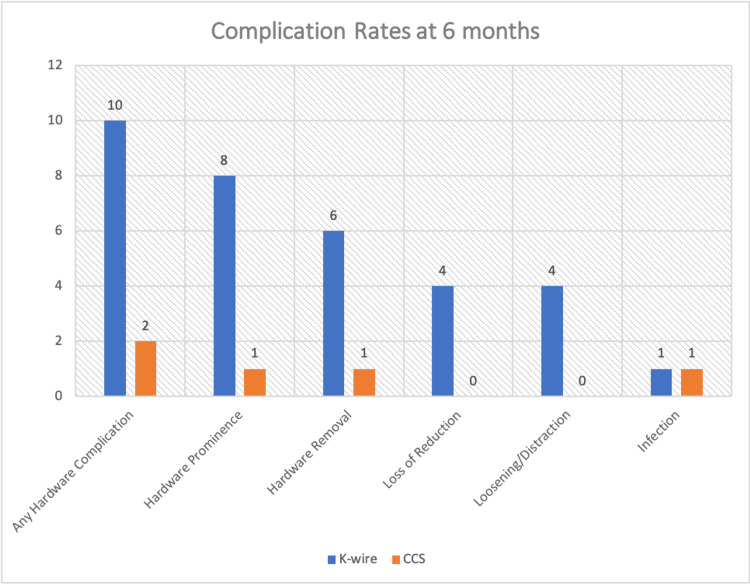
Comparison of complication rates at six months between K-wire and CCS fixation groups K-wire: Kirschner wire; CCS: cannulated cancellous screw

Results are depicted as a bar chart in Figure 4.

Superficial infection rates were similar between groups (5% each, p=1.000).

Return to Activity and Patient Satisfaction

Return to pre-injury activity levels was significantly higher in the CCS group (85% vs 65%, p=0.021). Patient satisfaction rates showed significant differences, with 95% of CCS patients reporting excellent or good satisfaction compared to 75% in the K-wire group (p=0.042).

Radiological Outcomes

At six months, complete radiological union was achieved in 90% of CCS patients versus 80% of K-wire patients (p=0.661). Mean time to radiological union was not significantly different between groups (CCS: 11.2±2.1 weeks vs. K-wire: 12.4±2.8 weeks, p=0.142).

## Discussion

In this trial, the CCS group achieved statistically higher Böstman scores compared with the K-wire group at six months (28.40 vs. 27.50, p=0.032). However, the absolute difference of 0.90 points on a 30-point scale did not reach the prespecified minimal clinically important difference (MCID) of three points. This indicates that while the functional difference was statistically detectable, it is unlikely to translate into a meaningful improvement in patients’ daily activities or overall quality of life. Thus, the functional outcomes of CCS and K-wire fixation at six months can be considered broadly comparable [15,16]. The clinical significance of our findings lies more in the substantial reduction in hardware-related complications, pain, and the need for implant removal in the CCS group, which represents outcomes of greater relevance to patient comfort, satisfaction, and healthcare utilization.

The most compelling findings relate to the dramatic reduction in hardware-related complications with CCS fixation. The fivefold reduction in overall complications (10% vs. 50%) represents a clinically and economically significant advantage [17]. This finding aligns with recent meta-analyses demonstrating high complication rates with traditional metallic fixation methods [18].

The superior early functional recovery in the CCS group likely reflects the biomechanical advantages of interfragmentary screw compression by translating biomechanical advantages into clinical benefits. The ability to recess screw heads below the anterior cortex while maintaining the tension band principle through cannulated design eliminates hardware prominence while providing stable fixation [19,20].

Early recovery benefits

The significant differences in early pain scores and functional recovery (two weeks to three months) translate into meaningful patient benefits. The CCS group's superior early recovery facilitates earlier return to work and activities, potentially reducing overall healthcare costs and improving quality of life [10].

Long-term implications

The sustained functional advantages and reduced complication rates at six months suggest these benefits persist beyond the acute recovery period. The 20% absolute difference in return to pre-injury activity levels represents a clinically meaningful outcome that impacts patient quality of life.

Comparison with contemporary literature

Our findings align with recent studies comparing these fixation methods. Mazyon et al. reported superior Lysholm scores with CCS fixation at three months [2]. Tian et al. demonstrated reduced complications and superior fracture reduction with cannulated screw constructs [8]. However, our study provides the most comprehensive six-month outcome assessment with validated functional measures and complete complication reporting (Table 3).

**Table 3:** A table comparing K-wire vs. cannulated screw tension band fixation in transverse patella fractures CCS: cannulated cancellous screw; K-wire: Kirschner wire; VAS: Visual Analog Scale (pain)

S.No.	Study & Year	Sample Size (K wire/CCS)	Follow-up	Primary Outcome (Score)	K-wire Result	CCS Result	Complications (K-wire/CCS)	Hardware Removal (K-wire/CCS)	Return to Activity (K-wire/CCS)	Key Conclusion
1	Current study (2025)	20/20	6 months	Bostman (max 30)	27.5 ± 3.15	28.4 ± 2.65	50%/10%	30%/5%	65%/85%	CCS is superior for function, pain, and fewer complications
2	Mazyon et al. (2023)[2]	11-Nov	3 mo	VAS (pain was lower and better)	3.92 ± 1.0	2.15 ± 0.7	Not detailed	Not detailed	Not detailed	CCS lowers pain, better function
3	Chiang et al. (2011) [7]	40 (K/CCS)	12 mo	Complications	62.5%/0% (loosening)	47.5%/ 5% (impinge)	62.5%/0% (loosening)	Not detailed	Not detailed	CCS has fewer hardware issues
4	Tian et al. (2011) [8]	52 (K-wire/CCS+Cable)	12 mo	Complications	15% loosening, 10% impingement	0% loosening, 0% impingement	25%/0% (loosening/impingement)	Not detailed	Not detailed	CCS+Cable have fewer complications
5	Wang et al. (2014) [9]	38 (K-wire/CCS)	12 mo	Complications	24%/0% (loosening)	13%/0% (impinge)	24%/0% (loosening)	Not detailed	Not detailed	CCS has fewer complications
6	Shrestha et al. (2020) [13]	20/20	6 mo	Complications	5%/0% (loosening)	20%/0% (impingement)	10%/5% (infection)	Not detailed	Not detailed	CCS has fewer complications
7	Liu et al. (2020) [15]	30/30	6 mo	VAS (Pain, lower better)	6 → <1 (1 mo → 6 mo)	4 → <1 (1 mo → 6 mo)	Not detailed	Not detailed	Not detailed	CCS had lower pain at all timepoints

The complication rates in our K-wire group (50%) align with published literature reporting high rates of symptomatic hardware requiring removal [18,2]. Mechanical complications were infrequent in the CCS group (10%), consistent with biomechanical studies demonstrating superior construct stability [21,22].

The substantial reduction in hardware removal requirements (30% to 5%) has important implications for healthcare economics and patient morbidity. This represents a seven-fold reduction in secondary procedures, significantly impacting both cost and patient experience [23].

**Figure 5:**
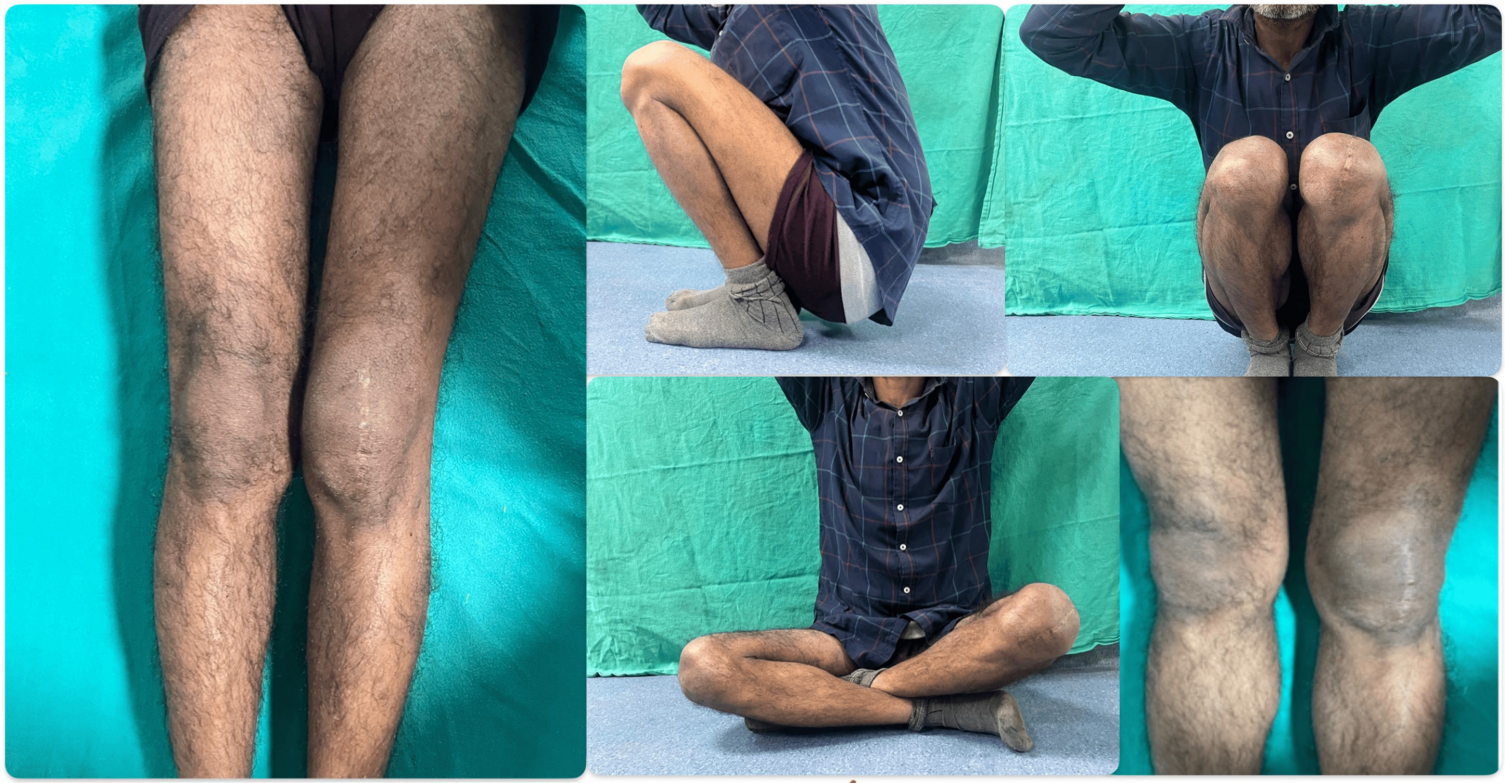
Six-month postoperative outcomes of the patient operated on with cannulated cancellous screws and tension band wiring

**Figure 6:**
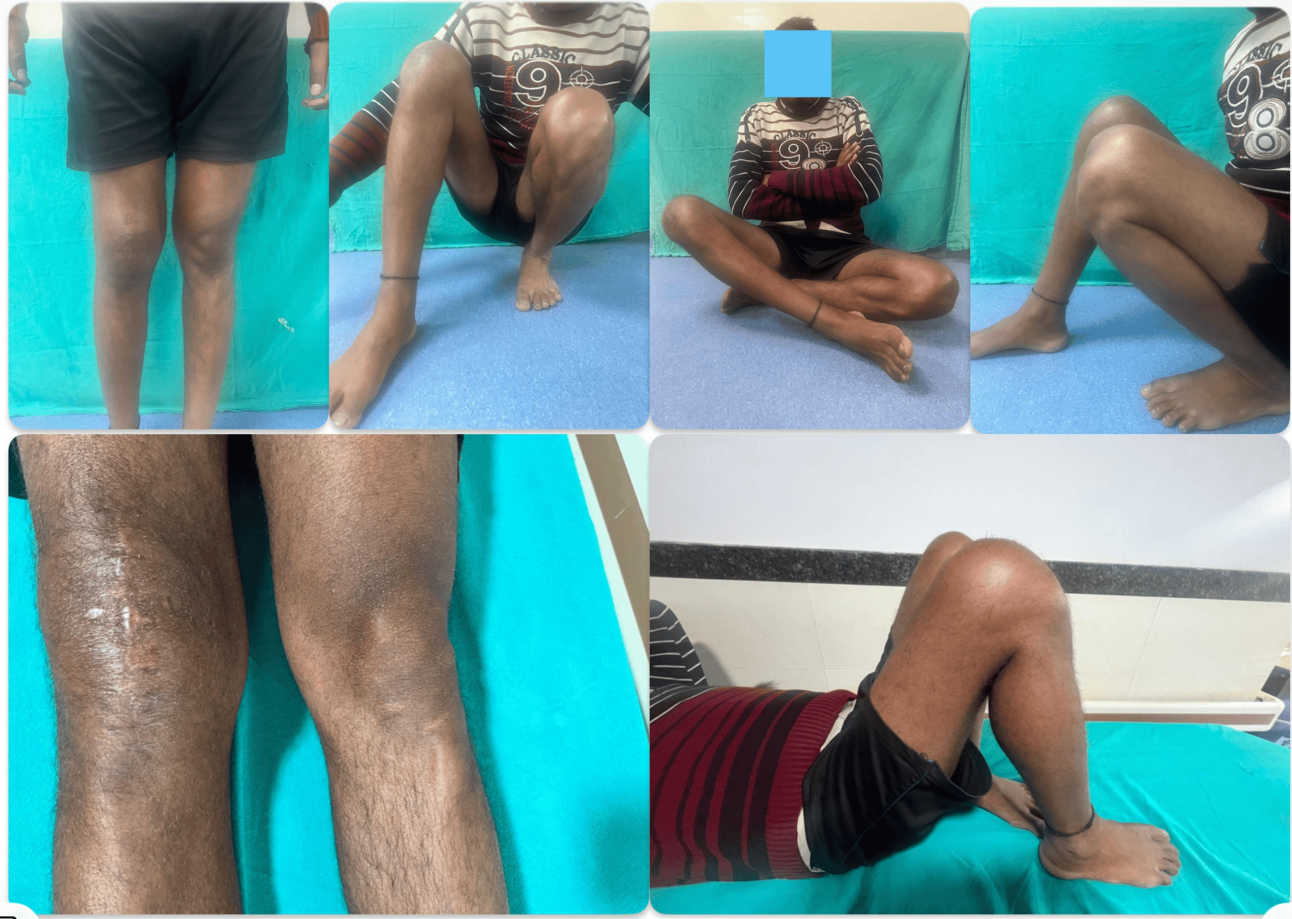
Six-month postoperative outcomes of the patient operated on with Kirschner wire and tension band wiring

**Figure 7:**
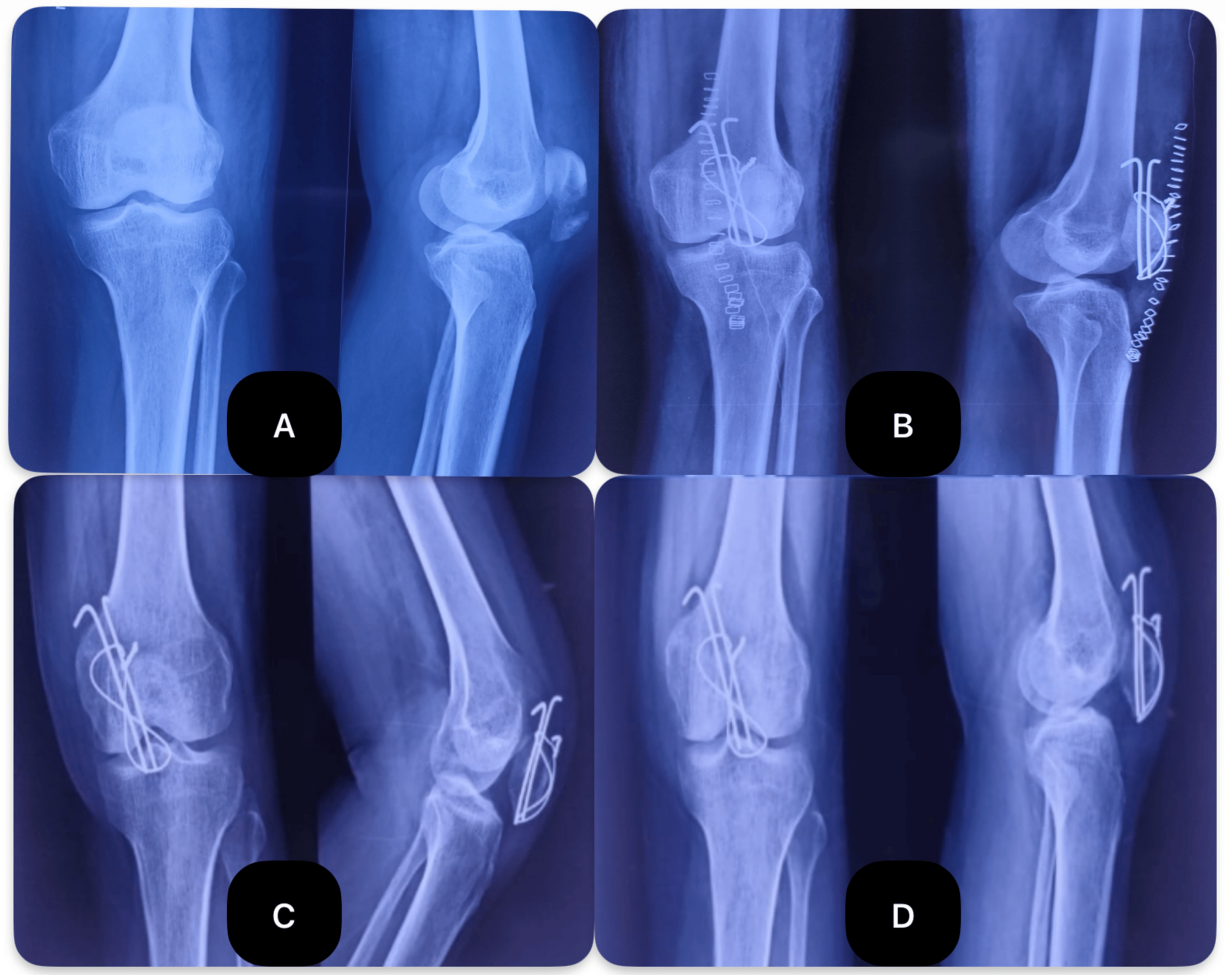
Preoperative and postoperative X-rays images of a patient operated with Kirschner wire and tension band wiring (A) Preoperative X-ray; (B) at two weeks postoperative; (C) three months postoperative; (D) six months postoperative

**Figure 8:**
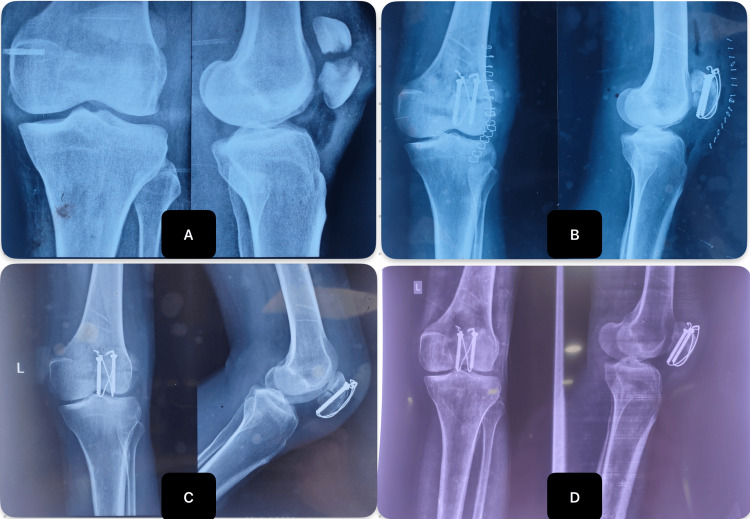
Preoperative and postoperative X-rays images of a patient operated with cannulated cancellous screws and tension band wiring (A) Preoperative X-ray; (B) at two weeks postoperative; (C) three months postoperative; (D) six months postoperative

These findings support the preferential use of CCS tension band fixation for transverse patella fractures. While the CCS group achieved slightly higher functional scores at six months compared with the K-wire group, the absolute difference (0.90 points on a 30-point scale) did not reach the prespecified minimal clinically important difference of three points, indicating no meaningful difference in final function. The major advantages of CCS fixation were its lower rates of hardware prominence, implant removal, and other complications, together with improved patient comfort and satisfaction. Thus, CCS fixation appears preferable primarily due to its safety and complication profile rather than a major functional benefit. Figures 5-8 show clinical-radiological images of both groups at various timelines.

Study strengths and limitations

Strengths

This study employed rigorous methodology according to CONSORT Outcomes 2022 guidelines [4], including proper randomization, allocation concealment, blinded outcome assessment, and comprehensive outcome reporting. The six-month follow-up provides adequate assessment of intermediate-term outcomes, and the complete retention rate eliminates bias from differential loss to follow-up.

Limitations

The single-center design may limit generalizability, though standardized surgical techniques enhance validity. The six-month follow-up, while adequate for assessing complications and functional recovery, may not capture long-term outcomes such as post-traumatic arthritis. The sample size, while adequately powered for the primary endpoint, may have been insufficient to detect smaller but clinically meaningful differences in some secondary outcomes.

## Conclusions

This prospective randomized controlled trial demonstrates that CCS tension band fixation provides superior functional outcomes, significantly reduced complications, and improved patient satisfaction compared to traditional K-wire fixation for transverse patella fractures at six months. The reduction in hardware-related complications, along with slightly better early functional recovery, suggests CCS fixation may be preferable for selected transverse patella fractures.

While the primary functional outcome difference was modest, the constellation of secondary benefits, including reduced pain, improved ROM, fewer complications, and higher patient satisfaction, provides compelling evidence for the superiority of CCS fixation. These findings have important implications for surgical decision-making and patient counseling in patella fracture management.
